# The Association of Diuretics and Proton Pump Inhibitors With Chondrocalcinosis

**DOI:** 10.1002/acr2.11260

**Published:** 2021-05-02

**Authors:** David T. Felson, Gabriela Rabasa, Xiaoyang Chen, Michael LaValley, S. Reza Jafarzadeh, Cora E. Lewis, James Torner, Michael C. Nevitt, Devyani Misra, MOST Study Investigators

**Affiliations:** ^1^ Boston University, Boston, Massachusetts, and National Institute for Health Research Manchester Biomedical Research Centre Manchester University Hospitals National Health Service Foundation Trust and The University of Manchester Manchester UK; ^2^ Boston University Boston Massachusetts; ^3^ The University of Alabama at Birmingham; ^4^ The University of Iowa Iowa City; ^5^ University of California San Francisco; ^6^ Beth Israel Deaconess Medical Center Boston Massachusetts

## Abstract

**Objective:**

Hypomagnesemia increases the risk of chondrocalcinosis and calcium pyrophosphate disease. We examined whether the use of drugs that can cause hypomagnesemia, diuretics and proton pump inhibitors (PPIs), increases the risk of chondrocalcinosis.

**Methods:**

Participants in the Multicenter Osteoarthritis (MOST) Study obtained weight‐bearing knee radiographs, and their medication use was recorded at baseline and 30‐, 60‐, 84‐, and 144‐month examinations. We read radiographs serially for chondrocalcinosis and characterized incident chondrocalcinosis when it first appeared. We classified diuretic use as thiazide, loop, and other. To test drug effects on incident chondrocalcinosis at each interval (eg, 30‐60 months), we excluded persons with chondrocalcinosis at the interval’s beginning. For each drug, we evaluated exposure at the beginning and end of the interval. We conducted knee‐based analyses using Bayesian mixed‐effects discrete time survival models adjusted for age, sex, body mass index, radiographic osteoarthritis, race, and clinic site.

**Results:**

Of 5272 knees, 196 developed chondrocalcinosis. Thiazide use (21.7% of examinations) and PPI use (13.7%) were common. Neither loop nor other diuretic use was associated with incident chondrocalcinosis. Thiazide use at the beginning and end of the interval of incidence conferred a high risk (hazard ratio [HR] = 2.18; 95% confidence interval [CI] 1.23‐3.89), but use at the beginning of the interval was not associated with risk (HR = 1.04). PPI use at the interval’s beginning increased risk of chondrocalcinosis (HR = 2.29; 95% CI 1.37‐3.79).

**Conclusion:**

Thiazide diuretics, but not other diuretics, and PPI use probably increase the risk of chondrocalcinosis. These findings may have important clinical implications.

## INTRODUCTION

Studies suggest that the radiographic prevalence of chondrocalcinosis—evidence of deposition of calcium crystals in the cartilage, which are usually signs of calcium pyrophosphate (CPP) deposition—occurs in 3.2% of knees of those aged 65 to 69 but increases to 27% of knees of persons age 85 and older (1). Its prevalence is increasing because of the aging of the population. Chondrocalcinosis is a radiographic finding, but the crystals that cause the calcification on x‐ray can induce painful pyrophosphate arthropathy or CPP disease.

Pyrophosphate is the precursor of CPP crystals. Because magnesium may act as a cofactor for pyrophosphatases, which break down pyrophosphate, high magnesium levels protect against the deposition of these crystals. Hypomagnesemia is an important risk factor for CPP disease, and an association of Gitelman syndrome (a genetic disease that predisposes to renal loss of cations) (2) with CPP is thought to be caused by hypomagnesemia.

Although chondrocalcinosis is often asymptomatic, there are few effective treatments for pseudogout. Nonsteroidal anti‐inflammatory drugs, although effective, are contraindicated in many older patients, and colchicine, which is effective in preventing acute attacks of pseudogout, has side effects that often preclude its use. On the other hand, diuretics are commonly used in older patients because of the high prevalence of both hypertension and diseases that cause extracellular fluid accumulation. Diuretics are also associated with hypomagnesemia, and results from studies of their association with clinical CPP disease have been inconsistent. There have been no studies, to our knowledge, on whether diuretics are associated with crystal deposition, the precursor to the clinical disease.

Not all diuretics act similarly on magnesium in the kidney. Thiazide diuretics cause magnesium loss and hypomagnesemia through effects on the distal renal tubule, whereas loop diuretics handle magnesium in the ascending loop of Henle. Although thiazides are associated with low serum magnesium levels, loop diuretics have been reported (3) to be unassociated with abnormal magnesium levels. Other diuretics work mostly by enhancing potassium retention and should have no effect on magnesium.

Proton pump inhibitors (PPIs) are another popular class of drugs reported to cause hypomagnesemia. They reduce the acidification of intraluminal contents of the proximal gut, and magnesium transporters depend on an acid environment for optimal function (4). Like studies of diuretics, studies examining the relation of PPI use with CPP disease have shown conflicting findings (1, 2).

Using data from the Multicenter Osteoarthritis (MOST) Study, in which there were serial knee radiographs, we examined the association of different classes of diuretics and of PPIs with the development of chondrocalcinosis.

## MATERIALS AND METHODS

#### Study sample

The MOST Study is a National Institutes of Health–funded longitudinal observational study focused on symptomatic and radiographic knee osteoarthritis (OA) in a cohort of community‐dwelling older adults with or at high risk for knee OA (5). The study enrolled 3026 participants age 50 to 79 years from 2003 to 2006 at two clinical sites (Iowa City, IA, and Birmingham, AL). Information regarding the participants’ demographic, medical, and lifestyle information, as well as imaging, was collected at baseline. Participants were observed with repeated comprehensive examinations at 30, 60, 84, and 144 months.

At each examination, participants were asked to bring to the examination site a plastic bag containing all of the medications they had taken in the last 30 days, and the examiner wrote down the medication names. If the participant did not bring all their medications, the examiner would call them and have the participant read any additional medication names off the bottles. Examiners then matched the medication’s generic or trade names to a medication code database provided by the MOST Study coordinating center. If the medication did not match exactly one from the database, it was sent to the coordinating center, where it was coded. Diuretic use was commonly reported, and the specific medicine was coded. The medication codes were linked to a drug information database that identified the specific ingredients contained in the medication.

#### Anthropometric measurements (body mass index)

Weight was measured to the nearest 0.1 kg on a standard medical balance beam scale, and height was measured on full inspiration to the nearest 1 mm with a wall‐mounted Harpenden stadiometer by certified MOST Study personnel following a written protocol. Body mass index (BMI) was calculated as weight in kilograms divided by the height in meters squared.

#### Radiographic assessments

Weight‐bearing, semiflexed posteroanterior and lateral views of the knees were obtained at baseline and each examination according to the MOST Study radiograph protocol (6). Each of two readers interpreted and graded all radiographs according to the Kellgren–Lawrence grade, and if they disagreed, readings were adjudicated by a panel of three readers (6). Chondrocalcinosis was assessed by a senior musculoskeletal radiologist as present or absent in each knee at each time point. Interobserver reliability against another experienced reader for the presence or absence of chondrocalcinosis in a knee was κ = 0.80 (95% confidence interval [CI] 0.70‐0.88).

#### Serum magnesium measurements

Fasting blood was drawn from MOST Study participants at baseline, and as part of a different study, 982 participants had serum magnesium measured. We examined reported drug use and its association with magnesium levels in this subgroup of participants.

#### Outcomes

For the study of incident radiographic chondrocalcinosis, we excluded MOST Study participants who had prevalent chondrocalcinosis in at least one knee at baseline. We defined a knee as having incident chondrocalcinosis when it developed chondrocalcinosis for the first time during follow‐up.

#### Analysis plan

We studied three different classes of diuretics, each with potentially different effects on serum magnesium levels and on the kidney’s handling of magnesium: thiazides, loop diuretics, and other diuretics (most commonly potassium‐sparing diuretics). For each, the referent group was non–diuretic users. For thiazide diuretics, we examined two subgroups of participants on the basis of findings from one study (3) that suggested that these subgroups of thiazide‐only users have different magnesium levels than those on a combination of a thiazide and other diuretics (most commonly dyazide). We combined exposure information from each PPI and treated them as a class in testing exposure.

We designed our analysis on the basis of assessments at fixed intervals determined by the study examinations. Each interval (eg, 30‐60 months) was treated separately, with exposure to a drug defined on the basis of the beginning (and end) of the interval and the outcome being a new occurrence of chondrocalcinosis on the x‐ray obtained at the end of the interval. The analysis contained four such intervals (0‐30, 30‐60, 60‐84, and 84‐144 months). For each interval analysis, we examined drug class as a risk factor for incident chondrocalcinosis. If it developed, chondrocalcinosis would have done so between examinations, becoming visible on the follow‐up radiograph. Use of a drug in the examination before could have contributed but so could drug use in the interim between examinations, which would have been reported at the later examination. For each interval, we tested two different definitions of drug exposure: For one, we defined a person (and their knees) as exposed if they reported drug use at the beginning of the interval. For the second, we stratified exposure into two levels, one for if the person (and their knees) was exposed only at the beginning of the interval and the second for if they were exposed at the beginning and end of the interval.

For analysis, we used the assessment intervals listed above. We conducted knee‐specific analyses using Bayesian mixed‐effects generalized linear models with complementary log‐log link to approximate mixed‐effects discrete time survival models (7) that were adjusted for age, sex, BMI, presence of radiographic knee OA at the preceding examination, race, and clinic site. Regression coefficients from our fitted Bayesian generalized linear model with complementary log‐log link are identical to those of a proportional hazards regression model, with an added advantage of addressing interval censoring. The exponentiated coefficients are interpreted as a relative effect on the hazard of outcome occurrence without an assumption that the hazard function is constant within each interval (7). We adjusted for the correlation between the measurements in two knees of the same study participants or measurements across study visits by inclusion of random intercepts for persons as well as for study visits. Our Bayesian analysis used noninformative priors and was conducted in R. Our analysis, after we excluded persons at baseline who had chondrocalcinosis in either knee, focused on knee‐specific incidence of chondrocalcinosis. This may introduce collider bias (also called index event bias) (8), in that chondrocalcinosis in one knee increases the risk of incidence in the contralateral knee, irrespective of drug usage. To ensure that our results were not so biased, we also conducted a person‐specific incidence, in which incidence was defined at the first evidence in a person of chondrocalcinosis in either knee.

The study protocol was approved by institutional review boards at Boston University; University of California, San Francisco; The University of Alabama at Birmingham; and The University of Iowa.

## RESULTS

After excluding participants with baseline chondrocalcinosis in either knee, we studied 5272 knees. Participants at baseline were, on average, 62.5 years of age and mostly women with a mean BMI of 30.7 (Table 1).

**Table 1:** Description of MOST Study participants at baseline and at examinations for which they were at risk for incident chondrocalcinosis

	Value
Age at baseline, mean ± SD, years	62.5 ± 8.11
Sex, % female	60.15
BMI at baseline, mean ± SD	30.74 ± 5.97
No. of knees with any thiazide use at all examinations of incidence/No. of person‐knee examinations (%)	2930/13,533 (21.7)
No. of knees with loop diuretic use at incidence examinations/No. of person‐knee examinations (%)	724/13,533 (5.4)
No. of knees with other diuretic use at incidence examinations/No. of person‐knee examinations (%)	861/13,533 (6.4)
No. of knees with PPI use at examinations of incidence/No. of person‐knee examinations (%)	1852/13,533 (13.7)
n/N of knees with OA at baseline (%)	2241/5272 (42.52)
n/N of knees with incident chondrocalcinosis through 144 months (%)	196/5272 (3.7)

Abbreviations: BMI, body mass index; MOST, Multicenter Osteoarthritis; OA, osteoarthritis; PPI, proton pump inhibitor.

One hundred ninety‐six knees developed chondrocalcinosis over the 12‐year follow‐up. Of the follow‐up intervals (up to four intervals per knee), 2930 (21.7% of intervals) had exposure at the beginning and/or end of the interval to thiazides, 724 (5.4%) to loop diuretics, 861 (6.4%) to other diuretics, and 1852 (13.7%) to PPIs. Most participants on thiazides used them in pure form without taking a combination pill containing a thiazide. Approximately 4% of knees developed incident chondrocalcinosis during the follow‐up.

Mean serum magnesium levels at baseline were lower in those on thiazides than those not on them (1.85 vs. 1.90 mg/dl; *P* = 0.0006), whereas those on loop diuretics had similar magnesium levels (mean 1.90 mg/dl for loop users vs. 1.90 mg/dl for non–loop users; *P* = 0.72). Users of PPIs at baseline did not have different mean magnesium levels from nonusers (1.92 vs. 1.90 mg/dl; *P* = 0.17).

When we examined the relation of diuretic use with incident chondrocalcinosis (Table 2), we found that consistent users (those who took thiazides at both the beginning and the end of the follow‐up interval) were at increased risk (hazard ratio [HR] = 1.79; 95% CI 1.09‐2.93) and that this was true especially of those who took only thiazides and not a combination of thiazide‐containing diuretics (Table 2). Results for the exposure based on thiazide use at only the beginning of the interval of incidence were less clear‐cut (HR for any thiazide use = 1.04; 95% CI 0.4‐2.29). We found no association of loop or other diuretics with chondrocalcinosis.

**Table 2:** Association of drug class with risk of incident chondrocalcinosis

Drug use	Adjusted HR (95% CI)
Any thiazide use	
None at examination before the interval	Referent
All with use at examination before the interval	1.42 (0.90‐2.22)
At examination before the interval only	1.04 (0.40‐2.29)
At examinations before and after the interval	2.18 (1.23‐3.89)
Pure thiazide use	
None at examination before the interval	Referent
All with use at examination before the interval	1.39 (0.83‐2.25)
At examination before the interval only	0.76 (0.34‐1.56)
At examinations before and after the interval	1.79 (1.09‐2.93)
Thiazide combined with other diuretic	
None at examination before the interval	Referent
All with use at examination before the interval	1.27 (0.53‐2.91)
At examination before the interval only	2.38 (0.87‐5.29)
At examinations before and after the interval	1.30 (0.38‐3.88)
Loop diuretic use	
None at examination before the interval	Referent
All with use at examination before the interval	0.80 (0.27‐2.09)
At examination before the interval only	0.71 (0.13‐3.37)
At examinations before and after the interval	0.69 (0.16‐2.29)
Other diuretic use	
None at examination before the interval	Referent
All with use at examination before the interval	0.48 (0.10‐1.59)
At examination before the interval only	0.60 (0.40‐3.64)
At examinations before and after the interval	0.80 (0.50‐5.48)
Proton pump inhibitors	
None at examination before the interval	Referent
All with use at examination before the interval	2.29 (1.37‐3.79)
At examination before the interval only	3.45 (2.03‐5.67)
At examinations before and after the interval	1.39 (0.60‐2.94)

Note

HRs were adjusted for age, sex, body mass index, race, clinic site, and presence of radiographic osteoarthritis.

Abbreviations: CI, confidence interval; HR, hazard ratio.

PPI use was also associated with incident chondrocalcinosis (see Table 2). This was not so clear among those with consistent use before and after the incidence examination (adjusted odds ratio [aOR] = 1.39; 95% CI 0.60‐2.94) but was more evident when we examined exposure to PPIs before the incidence examination only (aOR = 2.29; 95% 1.37‐3.79).

Analyses examining whether these drugs affected person‐specific incidence of chondrocalcinosis showed the same results.

## DISCUSSION

We found that persons on thiazide diuretics, especially if taken consistently, had an increased risk of new‐onset radiographic chondrocalcinosis. Loop diuretics were not associated with risk of chondrocalcinosis. We also found that PPIs increased risk. PPIs have been tied to acute CPP disease in comparison with acute gout (9). This is, to our knowledge, the first time the association of specific diuretics and of PPIs with chondrocalcinosis has been reported. Although we did not have information on the clinical diagnosis of CPP disease, our findings have clinical implications, suggesting that thiazides and PPIs might aggravate chondrocalcinosis and its associated symptoms. Our findings suggest that physicians may wish to consider alternatives for thiazides and PPIs in their patients with CPP disease.

Although analysis of all MOST Study participants over time did not include a measurement of serum magnesium levels, we conducted a cross‐sectional analysis of magnesium levels at baseline in a substudy of 982 MOST Study participants. Those on thiazides had lower magnesium levels than those not on diuretics. There was no significant association of magnesium levels with the use of loop or other diuretics nor with use of PPIs. This substudy focused on MOST Study participants without OA at baseline, and results may not be generalizable to other samples. Although a few other studies have also reported no association of PPIs with magnesium levels, four different meta‐analyses have reported an association of PPIs with low magnesium levels and have shown that persons taking these drugs are at increased risk of hypomagnesemia (4).

Results of studies examining the effects of drugs we studied on clinical CPP disease have been inconsistent. Rho et al (10), in a large electronic medical record (EMR) database, reported an increased risk of CPP disease with loop diuretics but not with thiazides. Lee et al (9) compared persons with clinical CPP disease with those with gout cross‐sectionally and found a higher rate of loop diuretic use in those with CPP disease. Recently, Tedeschi et al (11), drawing data from a large EMR database, reported that thiazides, loop diuretics, and PPIs all increased risk of clinical CPP disease. On the other hand, Kleiber Balderrama et al (12), examining US Department of Veterans Affairs data and adjusting also for magnesium levels, reported paradoxically that PPIs and loop and thiazide diuretics all decreased risk of CPP disease. It is unclear why these study findings differ, but acute CPP disease may be difficult to study in large databases, especially if synovial fluid diagnosis is not consistently available. As suggested earlier, evidence of drug effects on the incidence of chondrocalcinosis may complement the data from these studies given that crystal deposition is a precursor to the clinical disease.

Thiazide and loop diuretics handle magnesium differently. Loop diuretics induce magnesium diuresis in the ascending loop of Henle by reducing the electrical luminal/cellular gradient, which is necessary for magnesium reabsorption there. Thiazide diuretics act in the distal tubule to enhance magnesium diuresis, an action that may be due to effects of thiazides on a luminal transporter of magnesium or to depletion of potassium (13, 14). As noted earlier, a large‐scale examination of magnesium levels in the Rotterdam Study revealed that those on loop diuretics had, paradoxically, a tiny increase in serum magnesium levels, whereas thiazide users had substantially depressed levels, as we found here (3). Animal studies have suggested that chronic use of thiazides increases risk of magnesium depletion compared with short‐term use, and this is a possible explanation for our differential findings regarding duration of use.

There is a long history of hypomagnesemia causing chondrocalcinosis and even CPP disease. There are genetic causes of low magnesium and acquired causes, including parenteral nutrition. Furthermore, magnesium supplementation has been reported to be effective in preventing CPP disease attacks (15).

As noted above, PPIs cause hypomagnesemia. Magnesium absorption is thought to be dependent on two proteins located on the apical membrane of intestinal cells, the transient receptor potential melastatin 6 (TRPM6) and TRMP7 (7).These proteins have a high affinity for magnesium absorption and play a major role in maintenance of magnesium balance during periods of decreased magnesium intake. An acidic intraluminal milieu increases TRPM activity, and PPIs, which effectively suppress acid production, reduce its activity (13).

Among the strengths of our study were its large numbers, prospective analysis, and first (to our knowledge) examination of incident chondrocalcinosis as an outcome. Also, we were able to adjust for important confounders, age, sex, BMI, and especially preceding radiographic OA, which can increase the risk of chondrocalcinosis.

There were important limitations to our study. First, we had a limited number of incident cases of chondrocalcinosis, so confidence bounds were often wide, leaving some associations uncertain. We suspect that our differential findings regarding drug exposures before and after the interval of chondrocalcinosis incidence may be explained by small numbers of cases. Although we studied new‐onset chondrocalcinosis, we did not confirm any diagnoses of pyrophosphate arthritis, a clinically diagnosed condition best confirmed with crystals seen in synovial fluid with concurrent pain.

In conclusion, we report that thiazide diuretics and PPIs, but not loop diuretics, increase the risk of chondrocalcinosis. This finding has relevance for mostly older persons who have a high prevalence of CPP disease who tend to be frequent users of these two classes of drugs.

## AUTHOR CONTRIBUTIONS

All authors were involved in drafting the article or revising it critically for important intellectual content, and all authors approved the final version to be published. Dr. Felson had full access to all of the data in the study and takes responsibility for the integrity of the data and the accuracy of the data analysis.

### Study conception and design

Felson, Misra.

### Acquisition of data

Lewis, Torner, Nevitt.

### Analysis and interpretation of data

Rabasa, Chen, LaValley, Jafarzadeh.
